# Improvement of individual learning with mentoring programs for first-year undergraduate students

**DOI:** 10.3389/fpsyg.2023.1046999

**Published:** 2023-03-15

**Authors:** Marián Queiruga-Dios, Alvaro Perez-Araujo, Carmen Romero de Ávila-Arias, Araceli Queiruga-Dios

**Affiliations:** ^1^Accompaniment Institute, Universidad Francisco de Vitoria, Madrid, Spain; ^2^Faculty of Law, Business and Government, Universidad Francisco de Vitoria, Madrid, Spain; ^3^Higher Technical School of Industrial Engineering, Universidad de Salamanca, Salamanca, Spain

**Keywords:** mentoring program, student-teacher relationship, first-year students, undergraduate students, higher education

## Abstract

**Introduction:**

This study presents a brief analysis of Spanish universities that promote mentoring programs with students. These mentoring programs are divided into different categories depending on the persons (faculty or students) involved in the process and their characteristics (for novice, senior, or international students). The case of the Universidad Francisco de Vitoria is presented, where first-year students from all undergraduate degrees are involved in an annual course where the core part is related to formal mentoring activities.

**Methods:**

This study analyzes undergraduate degree students' outcomes and results from 10 different degrees for a period of 4 academic years (from 2016–2017 to 2019–2020). This first analysis corresponds to students' activities and marks awarded on the assessment of the assigned mentoring tasks related to the competencies of critical thinking, proactivity, personal knowledge (with the objective of acceptance and improvement), and the ability to ask transcendental questions. Then, a reliable and valid survey, conducted every year to all senior students, was used to get feedback from students.

**Results:**

After a quantitative and qualitative analysis of students' results, it was noticed that they become more confident in their studies when they engage in mentoring-based courses and sessions, and this benefits their lives. All this information led to the improvement of the mentoring process.

## 1. Introduction

Some degrees and majors have a long tradition of mentorship. This is the case in health profession education, where mentoring relationships between faculty and apprentices directly influence the learning process and increase student satisfaction and self-learning with training (Dutton, 2003; Williams et al., 2004; Jokelainen et al., 2011; Sayan et al., 2019; Gruber et al., 2020).

### 1.1. Mentoring programs

The definition of mentoring relies upon three essential characteristics: mentors are more experienced than mentees, mentors provide individualized support based on mentees' learning needs, and mentoring involves an interpersonal relationship, engagement, and commitment (Abdullah et al., 2014; Cornelius et al., 2016). However, there is no agreement in the definition and conceptualization of mentoring (Jacobi, 1991; Crisp and Cruz, 2009; Gershenfeld, 2014; Law et al., 2020). As is well known, “mentor” originated in the Odyssey, where Mentor was the character responsible for guiding Odysseus' son, Telemachus. This was a one-to-one relationship between an experienced person (Mentor) accompanying, guiding, and giving assistance to a less-experienced person (Telemachus).

Recent research about mentoring and its contemporary definition, with relevance for education, was performed by Mullen and Klimaitis (2021). They found, through a literature review, that mentoring promotes the growth of the whole person through guidance, intensity, reflection, and regulated learning, but mentoring is different from other mechanisms such as coaching, induction, or training; it is different from therapy, a one-way street, a cure-all, or a one-time intervention to fix a problem. Mentoring never occurs in isolation (Ziegler et al., 2021). It is recognized that “*trust, values, respect, empathy, and control are all essential aspects for mentoring programs, in addition to a feeling of belonging and connectedness; and the relationship between a mentor and a mentee is unique*” (Mullen and Klimaitis, 2021, p. 21). Moreover, mentors and mentees learn together during the mentoring process while extending their goals and what they reach.

The first-year mentoring program at the Universidad Francisco de Vitoria (UFV) is part of a course called Personal Skills and Competencies (PSC). In the case of sophomore students, mentoring activities continue with the Education for Social Responsibility (ESR) course. These transversal courses are included in all undergraduate degree programs at this university, and all students must attend; they are mandatory courses (Queiruga-Dios, 2020). First-year students arrive at the university, and sometimes they have a passive role without motivation or a sense of responsibility for the interaction with lecturers or with their classmates (Erickson et al., 2009). One of the mentoring program goals at UFV is to promote critical thinking, which is considered vital for students. A well-designed instruction will make students acquire a set of cognitive skills and face several challenges of adult life in such a way that it ensures effective function in the current increasingly complex world. Novice students usually have little experience at university and in personal and professional development. They are not capable of acquiring and transferring personal skills to their lives, i.e., to out-of-classroom contexts (Tiruneh et al., 2014). Thus, the mentoring process is significantly correlated with students' results in terms of their behavior, their attitudes, their engagement, and their retention rates, and certainly, that program is beneficial for students (Law et al., 2020).

The UFV mentoring program is a “university-based mentoring,” i.e., it takes place on the university grounds, typically during regular faculty hours for 1 h each month, with a total of 6 h per course (Karcher et al., 2006). Due to the COVID-19 pandemic, this has changed during 2019–2020, and mentoring sessions were conducted as e-mentoring activities. The UFV as several universities reacted quickly to the COVID-19 global outbreak, and faculty continue with classes and activities to provide learning opportunities to their students (Marinoni et al., 2022). The international association of universities survey, published in May 2020, shows that 85% of higher education institutions in Europe were able to quickly move from in-person classes to online sessions (Crawford et al., 2020).

### 1.2. Teacher–learner relationship and its implications in the educational process

Before going in-depth into teacher and learners' relationships, a brief definition of interpersonal relationships is included. A relationship between two persons consists of interpersonal interactions between them. Individual behavior, dyadic relationship, and social environment are some characteristics of interactions. Apart from the interaction, some cognitive and affective components and mutual awareness are involved in the relationship (Robertson, 1996).

The relationship between faculty and students could be considered a special category of interpersonal relationships, and it could be studied from different points of view. However, all of them agree that it has a significant impact on the quality and effectiveness of teaching and learning (Tiberius et al., 2002). Furthermore, teachers have changed their role from disseminators of knowledge (transmission of knowledge from one person to another) to facilitators of learning (to help learners in their own personal learning process). The new educational paradigm has proposed a “I-Thou” relationship between the teacher and the learner and left the “I-it” relationship behind (Robertson, 1996). Clinchy et al. (1996) established an interesting comparison between teachers and bankers or midwives. A banker–teacher's task is to deposit contents in the student's head, while the midwife-teachers draw it out. The midwife-teachers are known to participate in students' learning process, assisting them and promoting the development of their own fresh ideas, i.e., giving birth to students' proposals and contributions and thus constructing a different system based on students' experience (Clinchy et al., 1996; Queiruga-Dios and Crespí, 2021).

A similar metaphor was established by the objectivist model of teaching and learning, where the distinctive feature of teachers is “transfer” or “shaping.” The transfer paradigm is related to the transmission process where little attention is given to students as individuals and to teacher–learner links (Tiberius et al., 2002). The teacher–learner relationship is a formal relationship that is constrained by social, educational, and institutional norms. Some of the features of this relationship are the vulnerability of the learner compared to the teacher and an imbalance of power between the teacher and the learner (Haidet and Stein, 2006). Moreover, learners' autonomy, which has special importance in the European common educational space, depends on their capacity and teachers' capacity to generate an adequate personal learning context to meet and dialogue, where personal capacities are developed. This allows students to move toward becoming responsible for their own learning (Xhaferi and Xhaferi, 2011).

The mentoring relationship can describe a faculty–student, professional–student or student–student relationship. Mentoring is widely accepted as a complex, multi-dimensional, and effective mechanism for positively influencing undergraduate students (Livingstone and Naismith, 2018; Law et al., 2020).

Undergraduate mentoring programs have different goals; some of them are conducted to help students in their capstone projects or their first steps in research, work-integrated learning, or courses where a tutor is needed. Other programs aim to support new students, or students from ethnic and racial backgrounds in science disciplines, women in physical sciences, mathematics, and engineering, or students who are the first in their families to go to university. Formal and informal programs vary in terms of mentor training, activity type, and mode of interaction between students and mentors (Lunsford et al., 2017).

This study is centered on faculty–student mentoring, which facilitates the transition to university for first-year students. These mentoring programs have a positive impact on students, improve performance, and benefit student persistence and retention, as well as student success (Fox et al., 2010; Law et al., 2020). On the other hand, the mentoring program established by the UFV includes the definition established by Nora and Crisp (2007). These authors propose a multi-dimensional mentoring program with four dimensions: psychosocial or emotional support (empathetic and active listening and acceptance of the mentee's feelings), career guidance (including mentee's goal setting), academic and program support (acquisition of necessary skills and knowledge), and the existence of a role model (enriching the mentor–mentee relationship) (Nora and Crisp, 2007).

During mentoring sessions, the competencies of critical thinking, proactivity, personal knowledge, and the ability to ask transcendental questions are developed. The mentoring program at UFV includes a theoretical part to understand what the competencies are because students are not usually familiar with these topics. Critical thinking competence is related to the “deep look,” i.e., how I look at myself, how I look at others, and how I look at reality. This is developed using a questionnaire on the key concepts and with an experience of looking a person in the eye for a while or looking and observing how a person acts for a couple of days.

Proactivity is the attitude by which the person takes responsibility for his/her life, adopting decisions and assuming the consequences thereof. Therefore, being proactive implies assuming responsibility for things to happen, always deciding what we want to do and how we are going to do it (Frankl, 1985). To work on proactivity is done through SMART (specific, measurable, assignable, realistic, and time-related) actions (Doran et al., 1981). A series of SMART actions are used to meet the objectives that each person proposes to him/herself. For example, if one person's goal is to stop smoking, his/her actions can be to exercise, eat healthily, consult a specialist to receive help, and so on. The proactivity competence is worked with the entrepreneurship competence by using the Personal Development Project (Crespí et al., 2022).

To work on personal knowledge, SWOT analysis is used. During a mentoring session, an activity called “what is important in my life” is proposed, which consists of making a list of the important things and putting a grade on it from 1 to 10. Another column is added with the grade, from 1 to 10, of the level of involvement, and both columns are compared. If the level of importance is different from the involvement, the coherence of actions is analyzed. Transcendental questions are worked through the question “who am I” with a reflection task.

To develop the mentoring program, several specific training activities are developed each year for tutors/mentors. They participate in mandatory training sessions twice a year. The UFV accompaniment institute provides mentors with the required information and training, and all mentors belong to this institute. Moreover, an online repository, continuously updated, is available to all mentors.

### 1.3. Objectives and research hypothesis

The main objective of this study is to give value to mentoring programs at the university level. This will be provided by the following research hypothesis:

1. Spanish universities offer a mentoring program to their students. These programs aim to help students in their university stay.
2. Mentoring programs in first-year courses provide students with the competencies of critical thinking, proactivity, personal knowledge, and the ability to ask transcendental questions.
3. The mentoring program proposed by UFV helps students in their personal development and skills acquisition.

## 2. Materials and methods

This study includes three different methods: An internet search for mentoring programs in all Spanish universities, an analysis of data obtained from 187 first-year students that participated in the mentoring sessions for 4 years, and the analysis of results from a questionnaire conducted on all students from the Universidad Francisco de Vitoria during the 2020–2021 academic year.

The search for mentoring programs in all Spanish universities aimed to know which programs are more commonly used. The conference of rectors of Spanish universities (CRUE in Spanish) is a non-profit association made up of 76 universities, 50 public and 26 private. Most of them are in the community of Madrid (15), followed by Cataluña (11), Andalucía (11), Castilla y León (8), Valencian community (7), and the rest of the communities with <4.

Concerning the analysis of the Universidad Francisco de Vitoria mentoring program, results were collected from marks obtained by first-year students attending the mentoring program. The sample corresponds to the students monitored by the authors. The quantitative analysis was made from grades that were calculated with the activities developed during the PSC mandatory course. During this subject, students used to attend sessions with their teacher/mentor, and for each session, they must develop an individual work that is evaluated by the mentor. Apart from these activities, students must attend theoretical classes, develop teamwork about one of the theoretical topics, and participate in a social activity during the course.

Finally, to obtain some results and conclusions from students who participated in mentoring programs during their undergraduate degree studies, an institutional questionnaire is conducted every year to all senior students that participated in mentoring activities to determine their perception of the quality of the mentoring program and thus to detect points that could be improved. This provides useful qualitative results about students' status after 2 or 3 years have passed since they attended mentoring sessions.

**Figure 1:**
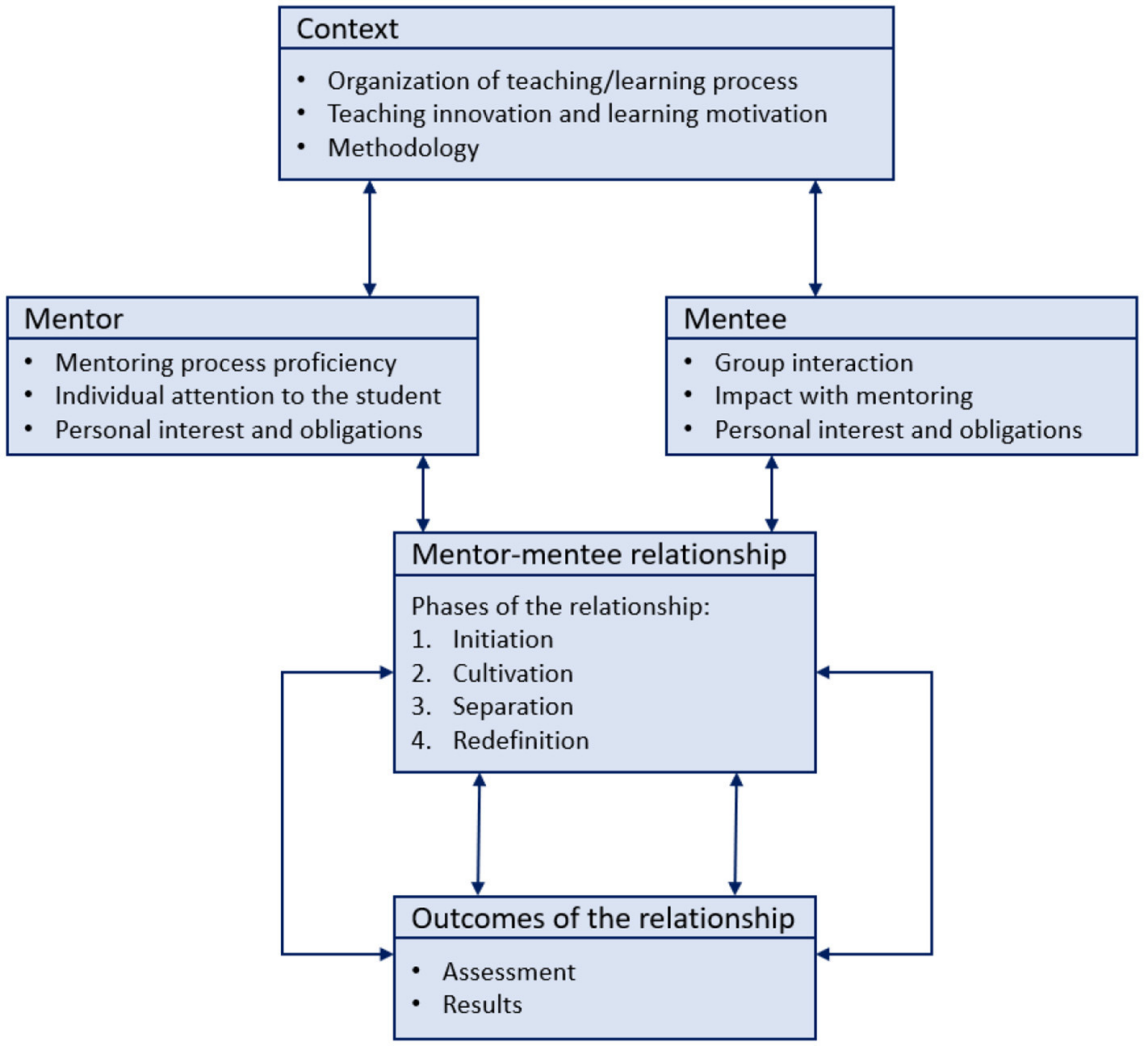
Mentoring process framework at UFV.

The UFV questionnaire consists of 25 elements, divided into two groups: 18 items about the mentor (organization, clarity in the exposition, innovative learning, motivation, interaction with the student, student attention, or results) and seven questions about mentoring process and assessment (methodology, impact, and training effectiveness). In each of them, a rating of 1–6 is requested to indicate the degree of agreement with the statements presented, considering that 1 is the lowest score and 6 is the highest. For this study, the following degrees were involved: Audiovisual Communication (AC), Business Administration and Management (BAM), Journalism (JOU), Computer Engineering (CEN), Criminology (CRI), International Relations (IIRR), Nursing (NUR), Fine Arts (FIA), Industrial Systems Engineering (ISE), and Law (LAW) degrees, 532 students participated in the survey. The distribution of questionnaire items is presented in Figure 1, where the mentoring framework proposed by Hunt and Michael (1983) was implemented at UFV. Moreover, the sequential stages provided by Kram are considered in the mentor-mentee relationship (Kram, 1985; Banerjee-Batist et al., 2019).

According to the ethical committee of the university, no ethical approval is needed because data are presented anonymously.

## 3. Results

### 3.1. Mentoring programs at Spanish universities

Mentoring programs are commonly used at Spanish universities; ~ 90% of these universities include some type of mentorship. The most common program in Spanish universities is aimed at students in their final year, and the less common mentoring program matches academics with first-year students. After searching for mentoring programs in Spanish universities, programs were divided into four groups:

- NOV: The mentoring program matches novice students that arrive at the university with third- and fourth-year undergraduate degree students.
- FORE: The Buddy program, the mentoring program, matches international students with local students.
- PROF: The mentoring program matches senior students and graduates with mentors, who are the university's alumni and professionals with relevant experience.
- TUT: The mentoring program matches academics with first-year students.

Of the 76 universities, eight do not include any information about their mentoring programs, so they were excluded from this study. The results of the search were as follows: NOV: 20, FORE: 19, PROF: 26, and TUT: 3. Several universities refer to their mentoring programs in different ways e.g., FORE programs such as the Buddy program, Mentor project, International Student Mentorship Program, International Host Mentor Program, and so on.

In the case of NOV programs, the following names were found: tutorial action plan, student mentoring program, tutor program, virtual welcome plan, student mentoring program, etc. A similar situation was found for the PROF programs, where the common names are related to the university, i.e., USAL mentoring program (from the Universidad de Salamanca) or Mentoring alumni UC3M (from the Universidad Carlos III in Madrid).

Of the 19 included in the FORE category, seven have also an NOV program, one adds NOV and TUT, and another one has a FORE program and a PROF program. In the case of NOV, two of them include a PROF program and another one, a TUT program. Finally, there are two universities that do not match these descriptions and have their own specific mentoring programs. This is the case of Universidad de La Laguna and Universidad de Málaga, with Comparte-ULL and GuíaME-AC-UMA programs, respectively. In the first case, PhD students train students from primary or secondary school, collaborating with the talent network International Mentoring Foundation for the Advancement of Higher Education (IMFAHE). GuíaME-AC-UMA is an enrichment program through university mentor workshops developed by researchers and PhD professors, aimed at students identified with high intellectual abilities from the 3^rd^ year of secondary education onward and coordinated and evaluated by specialists in Psychology and Education.

The three universities with TUT programs are private universities whose undergraduate degrees usually include courses related to ethics, social services, and so on, which is not common in public universities. One of these three universities is the Universidad Francisco de Vitoria, whose mentoring program was analyzed in this article.

### 3.2. Tutor–student mentoring program at UFV university

At the UFV, one of the core principles is to put the student (the person) in the center, which means that he/she will participate and be responsible for his/her own learning process. The mentoring program at UFV university is developed under this principle, which requires a different way of teaching, presenting trainers to the students, and proposing courses and programs that truly respond to the centrality of the person. First-year mentoring activities are divided into two areas: the community (in the classroom with the fellows) and the individual (with the teacher–mentor assigned to each student). During the mentoring sessions, the mentor and mentee walk together and go in depth through personal knowledge, the existence in the world, and where to go. At the same time, the mentee reflects and works on those topics being evaluated for it. As mentor-mentee meetings are individual, mentees find a confidential space in which they can share what they need to grow as a person, with confidence and a sincere interest in growing (Queiruga-Dios and Crespí, 2021).

During the first academic year at UFV, students participate in six individual sessions as part of their mentoring activities. The first part of these sessions is related to making the student know himself/herself. Once this is done, he/she must accept himself/ herself as he/she is. Finally, he/she will be able to surpass himself/herself. These are the three stages of the mentorship process.

From the sample of 187 UFV first-year students, Table 1 shows the number of students per degree (N), the mean and the standard deviation (SD) of the grades addressed by students from each degree (AC, BAM, JOU, CEN, CRI, IIRR, NUR, FIA, ISE, and LAW degrees).

**Table 1:** Descriptive statistics results from the complete set of marks of students from all degrees included in this study.

**Degree**	**N**	**Mean**	**SD**
AC	22	8.6000	1.22739
BAM	8	5.9896	2.66774
JOU	2	2.7917	3.94801
CEN	37	6.4126	2.59417
CRI	16	5.8479	3.32250
IIRR	37	7.3108	3.02272
NUR	52	8.0776	1.70522
FIA	9	6.5926	3.06709
ISE	2	8.3750	0.53033
LAW	2	2.9167	1.06066
Total	187	7.1978	2.63144

Comparing grades through the mean of the course obtained from all degrees, they are not significant because there is a big difference in the number of students. In this case, using an ANOVA one-way test, a *p*-value of 0 is obtained, which means that the null hypothesis (equal means) is rejected and there are at least two different means.

On the other hand, the distribution per year was analyzed, considering that in the last part of the 2019–2020 academic year (i.e., from March 2020), only online classes and activities were conducted. Due to the COVID-19 pandemic, there were no on-site activities from March 20^th^ until the end of the year.

**Figure 2:**
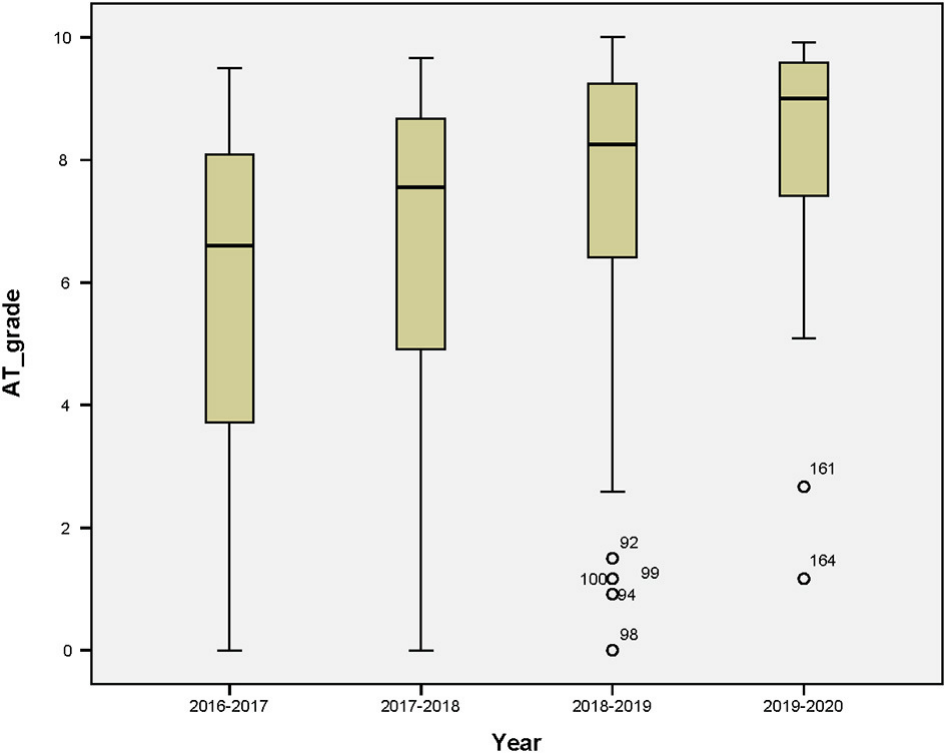
Boxplots of all data collected from 2016 until 2020 from students of several undergraduate degrees.

Figure 2 represents the boxplots corresponding to the final grades of the mentoring activities that were carried out from 2016–2017 to 2019–2020. There are seven outliers, five of them from 2018 to 2019 and two from 2019 to 2020, from IIRR and FIE, respectively (marks <3.0). These outliers correspond to students that get low marks. Sometimes this is due to their lack of motivation.

Table 2 shows the basic features of the data corresponding to descriptive statistical analysis. In this case, the academic year with the highest coefficient of variation is the first one (2016–2017), which has fewer students than the rest. This year has the highest skewness and the lowest kurtosis values. However, the more recent year (2019–2020) has the highest mean, median, mode, and kurtosis. For this, the range between values is the lowest one.

**Table 2:** Descriptive statistics from the data: The number of data (N), mean, median, mode, standard deviation (SD), coefficient of variation (CV), the lack of symmetry (skewness), the difference between the tails weight of the distribution and the tails of a normal distribution (kurtosis), the range, the minimum and maximum values, and the quartiles.

	**2016–2017**	**2017–2018**	**2018–2019**	**2019–2020**
*N*	15	60	65	47
Mean	5.730	6.581	7.354	8.238
Median	6.60	7.550	8.250	9.00
Mode	8.0	0.0	9.5	9.6
SD	3.1056	2.7027	2.5558	1.9063
CV	0.542	0.411	0.347	0.231
Skewness	−0.735	−1.015	−1.331	−1.872
Kurtosis	−0.649	1.69	1.031	4.007
Range	9.5	9.7	10.0	8.8
Minimum	0.0	0.0	0.0	1.2
Maximum	9.5	9.7	10.0	9.9
25%	3.667	4.875	6.417	7.417
50%	6.600	7.550	8.250	9.00
75%	8.167	8.708	9.250	9.583

### 3.3. Senior students' feedback about the mentoring program

Concerning the institutional survey conducted to get feedback from students, Table 3 shows detailed information about the students involved in it, separated by degrees.

**Table 3:** Distribution of the 532 students with degrees, including in this study participated in the final survey related to the mentoring program (2020–2021).

**Degree**	***N***	**Mean**	**Mode**	**SD**	**Skewness**	**Kurtosis**
AC	46	5.2892	5.00	0.244	−0.470	−0.226
BAM	67	5.2768	5.01	0.231	−0.248	−1.314
JOU	55	5.4192	5.24	0.240	−0.099	−1.239
CEN	41	5.4216	5.26	0.211	−0.305	−1.049
CRI	26	5.1468	5.12	0.0264	−0.293	−0.454
IIRR	30	5.3200	5.07	0.413	−0.190	−1.081
NUR	126	5.1816	4.90	0.232	−0.732	0.815
FIA	25	5.2120	5.40	0.298	−0.762	−0.062
ISE	61	5.4216	5.26	0.211	−0.305	−1.049
LAW	55	5.2280	5.07	0.287	−0.073	−0.969

Values of the skewness indicate that some cases, such as NUR and FIA, deviate from the symmetrical bell curve below the mean, lower than AC, BAM, and the rest of the grades. In the case of kurtosis, BAM and JOU get the biggest values, but essentially, all grades are quite similar in their distributions.

## 4. Discussion

Mentoring programs facilitate individual learning processes and strengthen students' personal and professional skills and competencies.

This study includes a basic statistical analysis of the results obtained by 187 students from 10 different undergraduate degrees, namely Audiovisual Communication, Business Administration and Management, Journalism, Computer Engineering, Criminology, International Relations, Nursing, Fine Arts, Industrial systems engineering, and Law. Moreover, the students' marks correspond to 4 academic years, from 2016–2017 to 2019–2020.

Some of the results obtained through the statistical analysis are as follows:

1. Means from different degrees are different. Apart from the difference due to the degrees with fewer students, the mean values of Audiovisual Communication and nursing are the highest. Both degrees have a common characteristic at the university; students that are involved in them have high grades in previous studies. They have good marks in pre-university exams.
2. The analysis by academic years showed that the highest values were obtained during 2019–2020.
3. During the COVID-19 pandemic, students feel comfortable attending mentorship sessions. They need to be in personal contact with tutors because this helps them in their daily tasks.

The authors have noticed that students are confident in their studies when they engage in courses such as Personal and Skills and Competencies and Social Responsibility.

Moreover, students' outcomes are sometimes not measured by good or bad marks in mentoring activities. In fact, some senior students have strongly appreciated the work and sessions with their mentors. When they reach the end of their stay at the university, they are able to appreciate what mentors have done for them. Some feedback was received from senior students about their first-year mentoring program. These activities helped them throughout their degrees and taught them to promote critical thinking, proactivity, personal knowledge, and the ability to ask transcendental questions, which are considered vital for students. They also developed transversal soft skills that are useful during their careers.

After mentoring activities, students are asked to fill in a survey to explain their appreciation and satisfaction with the program. They agreed that mentoring favors their learning, helps them in their daily lives, and encourages them to learn and to be in contact with their fellows. These are some comments from students following their participation in the mentoring program:

- This program helps to a great extent on a personal level, and I see the importance of the course (related to mentoring).
- Mentoring helps me grow as a person.
- With mentoring sessions, I learnt about myself; I learnt the meaning of proactivity and how to work on it. [SIC]
- Sometimes the workload is a bit heavy.
- Mentoring has been a space where I have been able to express and understand why I am the way I am.
- The development of mentoring is very good, they are dynamic activities, and if you know how to take advantage of them you can learn a lot since they are easy to apply in everyday life.
- I really liked the learning process with the mentoring sessions since they have given me the opportunity to have another perspective of those things that make me who I am. [SIC]
- Mentoring is very useful to me, and it gives me a good perspective on the future.
- I consider that the mentoring could be carried out in all the courses of the degree since they are a great help to us.

Mentoring activities developed at the UFV are proven to improve students' confidence and competencies. These activities, which have been part of the mission, vision, and values of the university since its foundation, lead authors to think about their benefits. To work on critical thinking, proactivity, personal knowledge, and the ability to ask transcendental questions to help graduates in their careers and motivate students during their stay at the university.

The mentoring program followed by the UFV accompaniment institute meets the requirements of good practices established by Campbell (2010). Thus, it is a formal and well-structured program, mentors are recruited and trained to provide a proper interaction with the protégés, and the frequency of mentoring sessions is fixed in the student's schedule. Moreover, as recommended by Law et al. (2020), the program includes strong psychosocial support and career and academic guidance.

## 5. Conclusion

The analysis of Spanish universities' mentoring programs shows that several universities have mentoring programs. However, the less common program is a mentorship between teachers (as mentors) and students (as mentees or protégés).

The multi-dimensional mentoring program proposed at the Universidad Francisco de Vitoria is built on four pillars: psychosocial or emotional support, career guidance, academic and program support, and the existence of a role model. This study shows the success of the mentoring program implemented at the UFV. Thus, mentoring activities improve students' life during their university studies. This program provides students with the competencies of critical thinking, proactivity, personal knowledge, and the ability to ask transcendental questions. Moreover, the UFV mentoring program helps students in their personal development and skills acquisition. Students' feedback leads the accompaniment institute to modify and improve the program to adapt it to newcomer students. Some of the improvements are related to mentors training, recruitment of volunteer mentors, the adaptation of the mentoring sessions to students' schedules, a course in the university learning platform, and the proposal of a mentoring program for mentors, i.e., mentors become mentees and participate in sessions like students.

The feedback obtained from senior students indicates the benefits of the mentoring program in their acquisition of competencies. Students' feedback can be grouped by the competencies that are involved in the mentoring process. Thus, personal knowledge and growth are clearly identified by them; other students write about proactivity and the ability to ask transcendental questions thinking about their future. Finally, critical thinking led them to analyze facts and daily activities and to observe and argue to find a result.

## Data availability statement

The original contributions presented in the study are included in the article/supplementary material, further inquiries can be directed to the corresponding author.

## Author contributions

All authors listed have made a substantial, direct, and intellectual contribution to the work and approved it for publication.
